# Rare Detection of Occult Hepatitis B Virus Infection in Children of Mothers with Positive Hepatitis B Surface Antigen

**DOI:** 10.1371/journal.pone.0112803

**Published:** 2014-11-10

**Authors:** Yong Liu, Jian Wen, Jie Chen, Chenyu Xu, Yali Hu, Yi-Hua Zhou

**Affiliations:** 1 Department of Laboratory Medicine, Nanjing Drum Tower Hospital, Nanjing University Medical School, Nanjing, China; 2 Department of Obstetrics and Gynecology, Zhenjiang Fourth People's Hospital, Jiangsu, China; 3 Department of Obstetrics and Gynecology, Nanjing Drum Tower Hospital, Nanjing University Medical School, Nanjing, China; 4 Department of Infectious Diseases, Nanjing Drum Tower Hospital, Nanjing University Medical School, Nanjing, China; 5 Jiangsu Key Laboratory for Molecular Medicine, Nanjing University Medical School, Nanjing, China; CRCL-INSERM, France

## Abstract

The prevalence of occult Hepatitis B virus (HBV) infection in children was considerably varied from 0.1–64% in different reports. In this study we aimed to investigate the prevalence of occult HBV infection among the children born to mothers with positive hepatitis B surface antigen (HBsAg) in Jiangsu, China. Serum samples were collected from 210 children of 207 mothers with positive HBsAg. HBV serological markers were detected by ELISA and HBV DNA was detected by nested PCR. Homology comparison of HBV sequences recovered from the child and mother was used to define the infection. Three children (1.43%) were positive for HBsAg, in whom the HBV pre S and S gene sequence in each child was identical to that in her mother. Of the 207 HBsAg-negative children, nine displayed HBV DNA positive by two nested PCR assays using primers derived from S and C genes. However, the sequence alignment showed that the sequences in each child were considerably different from those in his/her mother. Therefore, the sequences amplified from the children were very likely resultant from the cross-contaminations. Furthermore, the nine children with ‘positive HBV DNA’ were all negative for anti-HBc, and one had anti-HBs 3.42 mIU/ml and eight others had anti-HBs from 72 to >1000 mIU/ml, indicating that the nine children were less likely infected with HBV. Therefore, none of the 207 HBsAg-negative children of HBV-infected mothers was found to have occult HBV infection. We conclude that the prevalence of occult HBV infection in vaccinated children born to HBsAg positive mothers should be extremely low. We recommend that homology comparison of sequences recovered from the child and mother be used to define the occult HBV infection in children born to HBV infected mothers.

## Introduction

Hepatitis B virus (HBV) infection is a global health problem. Mother-to-child transmission of HBV is the main reason for the chronic infection in highly endemic areas [1]. Simultaneous use of hepatitis B immune globulin (HBIG) and hepatitis B vaccine in infants of mothers with positive hepatitis B surface antigen (HBsAg) has greatly reduced mother-to-child transmission of HBV [2].

However, it was recently reported that HBV DNA may be frequently detected in children with negative HBsAg, i.e., occult HBV infection, although the children were administered HBIG and/or hepatitis B vaccine. In 2009, Mu et al. reported that the rate of occult HBV infection was as high as 10.9% (5/46) among vaccinated children in Taiwan [3]. Surprisingly, as high as 28% and 64% of the vaccinated children born to HBsAg-positive mothers in India and Iran were respectively reported to have occult HBV infection [4], [5]. By contrast, other studies showed that the occult HBV infection rates were only 0.1–2% in Taiwan and African [6], [7].

Hepatitis B is endemic in China. It was shown that occult HBV infection among the young adults with positive hepatitis B core antibody (anti-HBc) from Qidong, Jiangsu, east part of China, was as high as 76.42% [8]. On the contrast, the rate in the children from north-east and north-west parts of China was only 0.77% and 4.92% respectively [9], [10]. Obviously, the substantial variations from different reports indicate that the true rate remains to be determined. The high rate of occult HBV infections in HBsAg-negative children appears to be unusual because vast majority of HBV infection manifests HBsAg positive as a result of the unique characterization of HBV replication [11]. Here, we report that occult HBV infection scarcely occurs in HBsAg-negative children of HBV-infected mothers by DNA sequencing and phylogenetic analysis.

## Materials and Methods

### Study subjects

Serum samples from 210 children (3 pairs of twins) born to 207 mothers with positive HBsAg during February 2008 to October 2012 were separately collected from two hospitals in Jiangsu, China, and the serum samples from mothers were also collected at the same time. All children were administered HBIG within 24 hours after birth and vaccinated with the recombinant HBsAg vaccine (Kangtai Biological Company, Shenzhen, China) on 0-, 1-, and 6-month schedule. The positive rate of hepatitis B e antigen (HBeAg) in the mothers was 39.61% (82/207). The children (male 112, female 98) were 2.5±1.6 years old. This study was approved by the Ethics Committee of Nanjing Drum Tower Hospital, Nanjing University Medical School, in accordance with the Helsinki Declaration. Each mother signed the written informed consent for herself and her child.

### Detection of serological markers for HBV infection

All serum samples from 210 children were measured for HBsAg and anti-HBc using commercially available ELISA kits (Huakang Biotech, Shenzhen; Kehua Bio-Engineering, Shanghai, China), and were quantitatively tested for antibodies against HBsAg (anti-HBs) with microparticle enzyme immunoassay (Architect system, Abbott, North Chicago), with positive ≥10 mIU/ml. When HBsAg was positive, the sample was further quantitatively tested for HBsAg, HBeAg, and anti-HBc with microparticle enzyme immunoassay (Architect system, Abbott).

### Detection of HBV DNA and sequence analysis

The serum HBV DNA was tested by nested PCR as our previously reported [12]. In brief, the DNA was conventionally extracted from 200 µl serum with proteinase K digestion and phenol-chloroform extraction method, and finally dissolved in 20 µl Tris-EDTA buffer. Nested PCR was performed using specific primers derived from the genes for HBV S, C and pre-C respectively as previously methods [12]. The low detection limit of the nested PCR with the three sets of different primers was each 20 copies/ml HBV DNA, equivalent to 4 IU/ml [12]. A diluted serum sample with final concentration of 4 IU/ml HBV DNA was always included in each PCR reaction as an internal positive control. All the positive samples at least by two PCR assays using different primers were also detected by the nested PCR using the primers derived from HBV reverse transcriptase (RT) gene; the external primer pair was 5′-TCCTGCTCAAGGMACCTCTA-3′ (sense, nucleotide 532–551) and 5′-AAACCCCARRAGACCCACAA-3′ (antisense, nucleotide 1017–998), and the internal primer pair was 5′-TGCACYTGTATTCCCATC-3′ (sense, nucleotide 593–610) and 5′-TGACAKACYTTCCAATCAAT-3′ (antisense, nucleotide 992–973). When the HBV DNA of the children was positive in the nested PCR, the HBV DNA from their mothers was also amplified with the same methods. To exclude the false positive caused by cross-contamination, we took particular precautions in performing each PCR step as suggested by Kwok and Higuchi [13]. The DNA extraction and each PCR round were undertaken in different rooms.

The samples in which HBV DNA was detectable at least by two PCR assays using different primers were tentatively considered to be positive. The resultant PCR products were purified and directly sequenced; the sequences were then compared with those amplified in this laboratory before and further compared with the sequence amplified from his/her mother. Only the sequence was different from other sequences but identical to mother's sequence, was the child defined to have occult HBV infection.

## Results and Discussion

In the present study, we measured HBV serological markers and HBV DNA in 210 children born to HBsAg-positive mothers. All children received combined passive and active immunoprophylaxis after birth. Three children (1.43%) were positive for HBsAg, HBeAg, and anti-HBc, and had HBV DNA 7.98×10^5^–2.41×10^7^ IU/ml (Table 1), demonstrating the HBV infection. Two of them were twins. All of the three children were born to HBeAg-positive mothers. Sequence analysis of the HBV pre S and S gene (1203 nucleotides) showed no mutations in the “a” determinant of HBsAg in the two mothers. The infected twins had same sequences between each other and between either of them and their mother, while another infected child showed the identical sequence to that in her mother, demonstrating the mother-to-infant transmission. On the other hand, 19 (1.6%) of the 1203 nucleotides (whole pre S and S gene) were different between the twin and another child, excluding the false positive caused by cross-contamination. Additionally, the high levels of HBsAg and HBeAg, and HBV DNA in each of the three children (Table 1) also indicated that they were really infected with HBV, rather than the cross-contamination. Phylogenetic analysis demonstrated that the three children were infected with HBV genotype B.

**Table 1:** HBV markers and alanine aminotransferase (ALT) levels in HBV-infected children and their mothers.

Sample	Mother					Child				
	Age (years)	HBsAg (IU/ml)	HBeAg (S/CO)	ALT (U/L)	HBV DNA (IU/ml)	Age (months)	HBsAg (IU/ml)	HBeAg (S/CO)	ALT (U/L)	HBV DNA (IU/ml)
D24L	26	76520	1286	22	4.32×10^7^	12	85090	1282	66.9	1.85×10^7^
D24S						12	159350	1408	59.9	2.41×10^7^
F51	31	49760	1168	25	9.2×10^7^	24	9690	922	253	7.98×10^5^

All three children were female, and children D24L and D24S were twins. Each subject was positive for anti-HBc. Phylogenetic analysis showed that all subjects were infected with HBV genotype B.

Of the 207 children with negative HBsAg, 164 (79.22%) had anti-HBs titers ≥10 mIU/ml, and 16 (7.73%), 18 (8.69%) and 9 (4.35%) had the antibody 5–9.9, 2.1–4.9, and <2 mIU/ml, respectively, while 8 (3.86%) were also positive for anti-HBc, indicating the resolved infection. Of these eight children, two had anti-HBs 8.92 and 29.48 mIU/ml respectively, and six others had anti-HBs >630 mIU/ml.

Since occult HBV infection may occur in seronegative subjects [14], we tested HBV DNA in all 207 HBsAg negative children, regardless of their anti-HBc status. We performed the nested PCR to amplify HBV DNA using three different primers derived from the HBV S, C, and pre-C genes, respectively. Nine sera displayed HBV DNA positive in the nested PCR with the primers derived from HBV S and C gene, respectively, but negative in the nested PCR with the primers from pre-C. These nine sera were further detected with nested PCR using the primers derived from the RT gene; none of the nine sera was positive. Nevertheless, based on the statements from the Taormina expert meeting [14], they might be diagnosed to have occult HBV infection.

**Figure 1:**
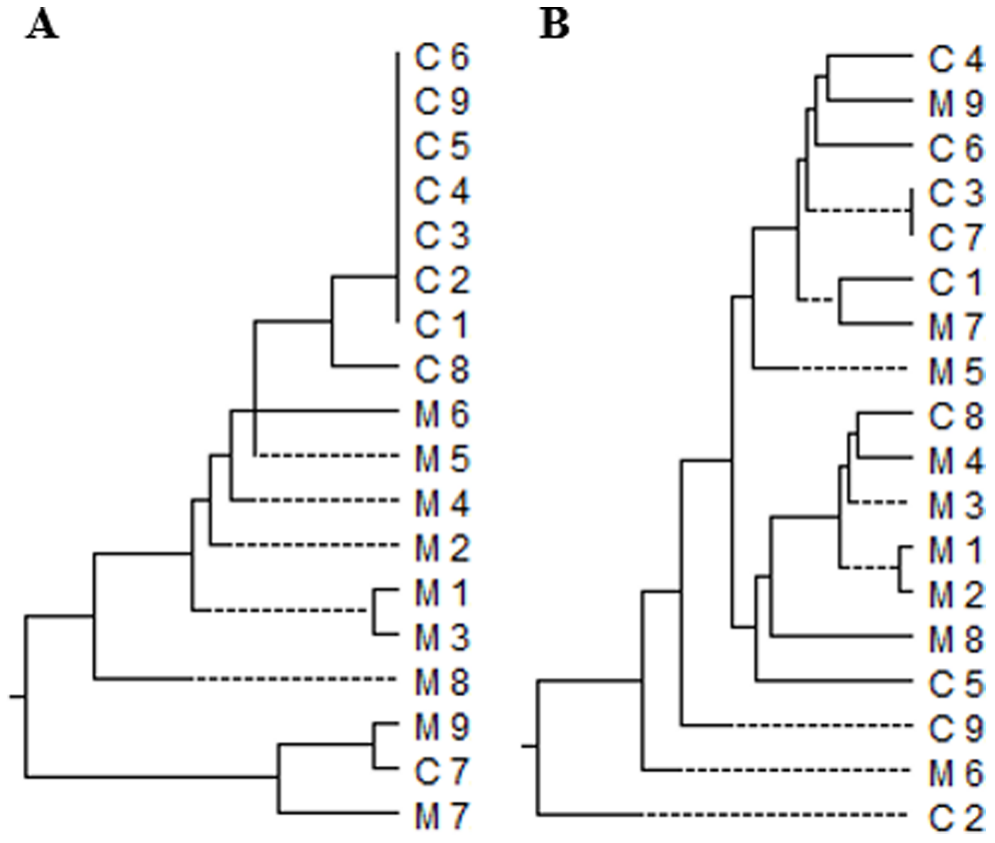
Phylogenetic analysis of HBV S and C genes in child/mother pairs. Phylogenetic trees were constructed based on the partial S gene sequences (A) and whole C gene sequences (B) from nine HBsAg-negative children with positive HBV DNA and their corresponding mothers. C: child; M: mother.

Since these nine children were 9–49 months old only and they scarcely contacted other members except for their HBsAg positive mothers and HBsAg negative fathers, we considered that these children's mothers should be the most likely infection source. Thus, we analyzed the S and C gene sequences from the children and their mothers. The sequence alignment analysis, including nucleotides 499–688 on the S gene covering the “a” determinant of HBsAg and nucleotides 2056–2283 on the C gene, showed that the S and C gene sequences in each child were considerably different from those in his/her mother (Figure 1). Moreover, the S gene sequences among the seven children were all identical, and two of the nine children had the identical C gene sequences. Additionally, of these nine children, one (4 years old) had anti-HBs 3.42 mIU/ml, eight others (0.8–3.6 years old) had anti-HBs from 72 to >1000 mIU/ml, but none of them was anti-HBc positive. Therefore, we considered the sequences amplified from the children were resultant from the cross-contaminations.

In the present study, the nested PCR may detect as low as 4 IU/ml HBV DNA, equivalent to one copy per reaction [12]. Thus, the nested PCR used in our study is highly sensitive. Furthermore, to exclude the potential ‘false negative’ in the PCR caused by the primer-template mismatches, the samples were separately detected by the three PCR assays using different primers of HBV genes and all the positive samples were detected by another primers derived from HBV RT gene. Therefore, it is less likely that the occult HBV infection in these children was not detected. On the other hand, we carefully collected the serum samples for detecting HBV DNA and took various preventive measures to exclude the false positive caused by cross-contamination [13]. To exclude the imperceptible cross-contamination, we compared the sequences from the positive PCR products and the sequences from their own mothers, and the known sequences detected in our laboratory. The molecular evidence (Fig. 1) demonstrates that the positive HBV DNA in these nine sera was caused by the cross-contamination. Based on the results that none of the 207 HBsAg negative children was defined to have occult HBV infection, we consider that the prevalence of occult HBV infection in vaccinated children of HBsAg positive mothers should be extremely low. Our results are comparable to the prevalence of occult HBV infection in vaccinated children to be around 0.1–2% [6], [7]. Elrashidy et al. also reported that HBV DNA was not detected in each of the 170 vaccinated children and adolescents in Egypt [15]. Therefore, the low rate of occult HBV infection may generally reflect the real situation. However, the low prevalence of occult HBV infection in our study is not consistent with other results, in which the rate was as high as 10–64% in the children despite prophylaxis with hepatitis B vaccination and HBIG [3]–[5].

Substantial variation of the reported occult HBV infection rates from the same regions causes concern of cross contaminations in the detection of HBV DNA. For examples, among HBsAg-negative adults in Soul, South Korea, HBV DNA was detected in up to 16% of the subjects in one report [16], but only 0.7% in another survey [17]. In Taiwan, the occult infection was reported to occur in 10% of the children [3], but only in 0.1% of the vaccinated children [7]. It is noticeable that the serum samples in either of the two reports with high occult HBV infections had been used for routine laboratory tests before the detection of HBV DNA [3], [16]. It is very likely the samples had carry-over contamination during the routine laboratory tests.

Since HBV is prone to undergo genetic mutations, the viral sequences from different individuals scarcely have identical or nearly identical nucleotide sequences except they were infected with the same infection source. Therefore, the homology of viral sequences may serve as molecular evidence for HBV transmission. We propose here that the sequence homology may also serve as convincing evidence to determine the cross-contamination when individual persons have not contacted each other or not contacted the same infection source. Therefore, the results that as high as 51.2–76.5% of the individuals with occult HBV infection had identical viral sequences [5], [8] were very likely caused by cross-contamination in laboratory testing. In our study, seven of the nine children with positive HBV DNA had the identical HBV S gene sequences, which indicated the occurrence of cross-contamination.

False positives are not rare events in PCR-based tests. Although precautious steps were proposed to avoid false positives soon after the development of PCR [13], imperceptible false positives may have occurred. It was reported that novel hemagglutinin-esterase gene of human torovirus had been cloned [18], but it was a result of cross contamination by another virus occurred at the inception of the process [19]. Similarly, hepatitis E virus was reported to be isolated from rodents [20], which turned out to be caused by cross-contamination [21]. Moreover, simian virus 40 was proposed to be associated with the development of non-Hodgkin lymphoma [22], but it was demonstrated to be associated with false-positive PCR results [23]. Additionally, in a chimpanzee experimentally challenged with plasma of a Japanese donor infected with hepatitis C virus (HCV), while the sequences of the hypervariable region 1 of HCV isolated weeks 3–14 after challenge were identical or high homology to the sequences in the donor, the E1 clone isolated week 16 after challenge was not detected in the original plasma, and then the virus was no longer detected [24]. However, Blaster analysis showed that the E1 clone is closely to the sequence of HCV-1 isolated in USA (accession No. M62321), indicating the existence of the probable cross-contamination, since the investigators in these two laboratories used to collaborate in the study. In the present study, the viral sequence amplified by PCR in each HBsAg-negative child was substantially different from that derived from his/her mother. Since these young children scarcely contacted other persons, we consider the viral sequences isolated in the children were resultant from the imperceptible cross-contamination.

In summary, occult HBV infection was determined in none of the 207 children born to HBsAg-positive mothers. We consider that the prevalence of occult HBV infection in vaccinated children born to HBsAg positive mothers should be extremely low. When occult HBV infection occurs in a child of HBsAg-positive mother, the mother should be the most likely infection source. Thus, we suggest that homology comparison of sequences recovered from the child and mother should be an important criterion to define the occult HBV infection in children born to HBV infected mothers.
